# Zero Watermarking: Critical Analysis of Its Role in Current Medical Imaging

**DOI:** 10.1007/s10278-020-00396-0

**Published:** 2021-01-07

**Authors:** Aleš Roček, Michal Javorník, Karel Slavíček, Otto Dostál

**Affiliations:** Institute of Computer Science, Masaryk University, Botanická 68a, Brno, 602 00 Czech Republic

**Keywords:** Watermarking, DICOM, Medical imaging, Security, Integrity, Copyright

## Abstract

Medical image data plays a critical role in health care today. Whatever the context of image processing (research and educational activities, diagnostic or forensic purposes, etc.), they are supposed to be treated as highly sensitive. Unfortunately, currently available image-processing tools also enable very sophisticated malicious modification of their content.

This paper is focused on the assessment of the effectiveness of selected so-called zero watermarking methods (ones that do not cause any modification of an image sample), in the protection of the integrity, and proving authorship, of these medical image studies. We have studied many zero watermarking methods and selected one representative from each type of known algorithms for comparison.

We have conducted a series of simulations over a huge research database of anonymized medical image studies (patient examinations).

## Introduction

Emerging networks of health professionals, medical communities, patients, organizations, and other stakeholders are reshaping regional radiology practices these days. The technologies being employed for global medical imaging enable the integration of common radiology concepts and the reconciliation of global radiology. New opportunities also bring new risks to this field.

Due to the easy distribution of multimedia data (for example, medical image data) via the Internet and the wide availability of powerful image-processing tools (ease of editing, duplication, altering, theft, etc.), it has become difficult for the authorized users (or owners) of image data to protect its originality, prove their ownership, prevent misrepresentation, unauthorized use, misappropriation, and so on.

Many new techniques based on watermarking have been introduced in the past few years. Current digital watermarking methods represent a good alternative to enforce many of the aforementioned rights. A critical review of some so-called zero watermark-based techniques, highly relevant for medical imaging, has been proposed in this paper.

There are two main fields of interest in digital medical image protection. The first one is the protection of originality, where the goal is to ensure or enforce originality of an image because images with unauthorized modifications cannot be used for diagnostic purposes. The second one is proof of authorship because specialists using medical images for scientific and educational purposes do not intend their work to be copied and reused without the authors’ permission.

Watermarking is not meant as a competitor of another DICOM image protection mechanisms like a digital signature but as a complementary one. The digital signature can ensure bit-by-bit conformity of two DICOM images, including image metadata. Watermarking protects the image part only, but on the other hand, it can provide more information than a digital signature. The digital signature provides binary output only—images (or more precisely files) are or are not identical. Zero watermarking can provide some similarity measure as well. In the case of composite images like CT, where the DICOM entity consists of many slices = images, each slice should be protected by watermark separately. This apparent disadvantage has one meaningful benefit: in case someone has modified just a few slices, e.g., slices depicting a tumor, watermarking will identify the modified slices. The price for this ability of watermarking is the necessity of storing as many watermarks as many slices we need to protect.

This paper analyzes the abilities of zero watermarking algorithms to serve as proof of authorship of medical images. Zero watermarking, in principle, seemed to be promising to achieve this objective. We have done intensive testing of several zero watermarking algorithms. The results are summarized in this paper.

## Regional Medical Imagining

Masaryk University, Czech Republic, develops and runs a system that provides new platforms for very efficient regional cooperation in the field of medical imaging. Currently, the system enables secure communication between hundreds of health care institutions, mostly from the Czech and Slovak Republics.

The communication capabilities of the system make it possible to transfer highly sensitive patient image data between different medical devices or applications located in health care institutions, which may be far from one another.

Supportive technology allows defined interactions among the external environments involved. The main stakeholders are specialized health care institutions, general and university hospitals, individual radiologists, universities and research centers, ICT services providers, etc. One of the key issues is the expression of authorization boundaries and the enforcement of access rights to distinguish those who may use individual datasets and those that must not.

An individual image examination (medical image study) generated by acquisition modalities, such as computed tomography, magnetic resonance, etc., is composed of a series of individual medical images. They include personally identifiable information (contact details, treatment details, etc.) Unauthorized disclosure or unauthorized modification of health-related data affects that particular patient (for instance, an inaccurate diagnosis) as well as the health care institutions who, according to regulatory laws, hold accountability for the data processing. The information security requirements can be expressed as the confidentiality, integrity, and availability of clinical data and related processes. The processing must also be in line with relevant legal, ethical, and contractual requirements. Those regulatory laws and policies are becoming very strict in most countries these days.

## Scenario of Using a Watermarking

The environment (processing) for which we evaluate the suitability of the watermarking schema can be defined as the processing taking place over a set of image objects originating from routine image examinations and retrieved from operational PACS systems of cooperating health care institutions. The selection of specific series or individual images from the initial imaging examination like CT typically disrupts attributed digital signature. There is a necessity to share highly sensitive data. The set of objects is shared (modified) within the community of legitimate users and controlled by its owner. The trust between the owner and other parties can be grounded on privacy regulations or research/business contracts.

The typical examples are cooperating research activities over clinical data, i.e., sharing the data with trusted third parties, where the integrity protection, authorship, and other security issues are of critical importance. Hospitals and medical research centers form a unified computerized environment these days where traditional diagnosis, treatment, and research activities intertwine.

Appropriate watermarking schema enables different kinds of information to be treated in different ways. It can encode the knowledge of the owner and the identity of the third (trusted) parties, and then, for instance, reveal the violating the illegal sharing with non-trusted parties. The supportive rules can be set up, then enforced via proposed watermarking schema, and this way prevent unwanted disputes over the source data, the integrity of its essential parts, protection of highly sensitive information attributed to the image, research results, etc.

Typical usage of the proposed watermark schema for the above-defined processing consists of some combination of the following steps:

- generation of the introductory watermarked image study (embedding the expanding information parts, patient ID, research ID, an ID of approved users, etc.)
- enabling legitimate participants to process the watermarked image study
- parts of the watermarked image study can be provided with additional information (modified) during the cooperating activities
- extraction of the hidden information from the watermarked image by authorized users
- evaluation of the quality of the extracted watermarks and, thus, the consistency of the intended processing (clinical trial, etc.)

The critical features of the proposed watermarking schema relevant to the above-defined usage scenario:

- prevention of unauthorized modification of regions of interest
- the ability to identify the conducted clinical study or identify patients who participate in the imaging study, integration with image information, avoiding separation
- the possibility of hiding required information
- the ability to provide the critical parts of an imaging study in its original quality.

## Zero Watermarking

A traditional digital watermark hides information about the owner or creator of an image or images somewhere within that image. This hidden information can subsequently be used for many purposes: maintaining the integrity of the image, detecting deliberate or accidental tampering, protecting copyright in the data, etc.

Regarding medical imaging, watermarking schemas such as zero watermarking, reversible watermarking, and lossless watermarking (focused on the so-called region of interest) in particular—as well as some of their interconnections—seem to be highly relevant. In most cases, the degradation of medical images is unacceptable due to the high image quality requirements of diagnostic processes. So-called zero watermarking can successfully solve these specific requirements.

The main advantage of the zero-watermarking method in medical imaging is that the watermark is not embedded in the image itself so, unlike traditional watermarking techniques, the watermarking process does not apply any modification to the image, thus avoiding any distortion of the image. Hidden features are extracted from the host image and combined with the watermark (some kind of hidden information, such as a logo) and then encrypted, and a key is produced. The secret share must then be kept in a trusted authority for future watermark extraction. So, the extraction of internal representative feature information from the image data is the critical phase in the zero-watermarking approach.

## Our Research

The zero-watermarking algorithms being reviewed were evaluated using samples taken from suitable datasets of medical image examinations. We especially focused on the detection of malicious tampering, such as the addition or removal of objects of interest from the critical medical image (which can be in some situations even life-threatening). Some medical image examinations (for instance, computed tomography or magnetic resonance) produce a series of very similar images. The crucial problem considered in this paper is how to solve the authorship issue in the case of very similar but different images (from different medical examinations or different patients) and the case of one image and its modified copy.

According to the current state of our research, it is practically impossible to find a suitable solution matching all the requirements and constraints relevant to this particular application area. We will only succeed if we focus on some particular services or applications, their specific features, and crucial security issues. Regarding data from medical image examinations, these issues are the authenticity and integrity protection of linked series of images.

Security issues such as accepting forged content as if it were legitimate represent one of the most serious problems in the area of medical image processing. This kind of consideration in particular shapes our research. In general, it is evident that to develop a perfectly fitting watermarking system is not possible. But another question to be resolved is an assessment of particular real scenarios (specific application requirements, relevant legal regulations, etc.) and their practical achievability (considering the available computational power of the equipment involved, the total complexity of the method, and so on).

## Methodology

The main goal of this paper was to answer the following questions. First, can an appropriate zero watermarking method be regarded as a suitable prospective one for checking the integrity of medical image examinations? Second, can an appropriate zero-watermarking method be regarded as a prospective one for checking the authorship of medical image case studies? Specifically, could suitable watermarking methods reveal some referential threshold to identify the required integrity or authorship changes?

From a variety of available medical image datasets, we have selected cross-sectional CT (computed tomography) scans of abdomens with diagnosed tumors.

CT scanners currently represent one of the busiest acquisition modalities. A typical CT examination, also called an image study, consists of hundreds or even thousands of individual slices (images), which are then organized into a specific series. Each image slice, depending on the type and configuration of the particular CT scanner, represents 1 to 10 mm of the patient’s tissue. Images are mostly displayed and diagnosed individually or used to generate a three-dimensional picture of the patient’s body. CT scans enable the efficient identification of diseases of the various body regions, such as revealing possible tumors. They display far more complex information compared with X-ray examinations.

All the medical image data necessary for this research was selected from the long-term archive of anonymized image examinations developed to support of specific research activities. Its content was originally gathered from the Clinical PACS (Picture Archiving and Communication System) of health care institutions from the region.

We have implemented four promising zero-watermarking methods. The testing was conducted over three types of datasets representing the likeliest adverse scenarios.

The first type represents the manipulation of critical medical content such as adding, removing, or replacing some of the critical parts of the tissue, organ, or tumor. We used a graphical editor to gradual substitute of tumor by copying nearby healthy tissue in the CT image. After removing whole tumor, we started removing firstly small organs and secondly large ones.

The second type represents the case of replacement of the key images in the series with nearest and most similar ones belonging to the same patient.

The third type is similar to the second one but utilizes appropriate images from the same part of the screening of another patient.

The resulting graphs visualize the computed similarity dependences of series of intentionally manipulated images and a series of relevant original ones. The images are arranged into sequences according to a reference PSNR (peak signal-to-noise ratio) metric [1].

Considering the selection of a particular acquisition method (CT) and selection of a particular disease (tumor), it is not possible to generalize the results about the appropriateness of investigated zero-watermarking methods into other domains of medical imaging.

## Zero Watermarking Principles

**Fig. 1:**
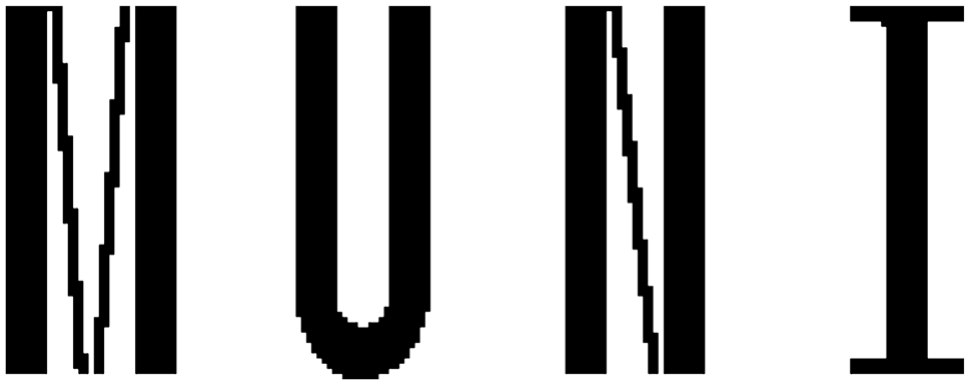
The inserted watermark

Necessary context, including related terminology, has been described in detail in [2]. The common principle of all zero-watermarking algorithms is straightforward: consider an original (for simplicity’s sake, a grayscale) image and a black and white watermark image, e.g., a company logo. We used the watermark shown in Fig. 1.

Using these two inputs (and optionally a secret key), we can compute two outputs: a public share and an ownership share. Nevertheless, the ownership share contains enough information to prove that it was computed from the original image and the watermark. The ownership share is used for internal computation only. It should be stored in a secure and trusted place, e.g., dated and signed by a public certification authority.

**Fig. 2:**
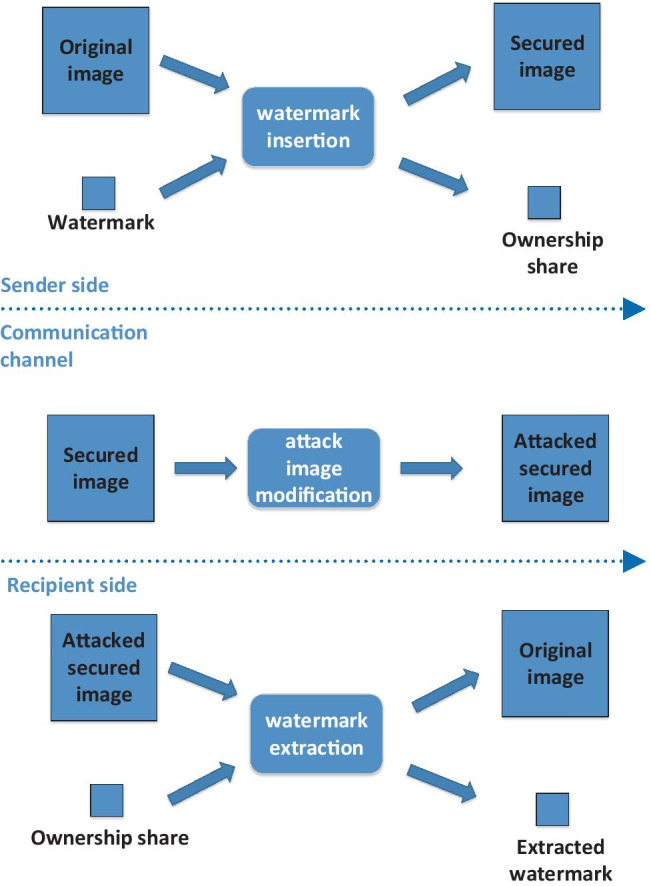
The principle of zero watermarking

Later on, the ownership share serves for image authorship approval. If we meet an image we suspect to be ours anywhere, we can check it with the following principle illustrated in Fig. 2.

The discussed watermarking algorithms differ in exactly how the ownership share is computed from the original image and the watermark. A detailed description of the main zero-watermarking algorithms is in the following subsections.

## Reference Zero-Watermarking Methods

For simulations and testing purposes, we selected four zero-watermarking methods with different security approaches. These methods represent the wide-scale state of the art in the field of zero-watermarking methods in medical imaging.

The first method, “Robust image watermarking scheme using visual cryptography in dual-tree complex wavelet domain”, uses as its input the original image, a watermark, and a secret key. [3]. For transformation and watermark insertion, *k-level Dual-Tree Complex Wavelet Transform* (DT-CWT) [4] is used. For proof of authorship, secret share, secret key, the level of transformation, and the original image (in our case modified image) are used.

The second method is “A Zero-Watermarking Scheme using Discrete Wavelet Transform” [5]. As an input, it uses the original image, a binary watermark, and four secret keys. The image is segmented and transformed by a 1-level discrete wavelet transform [6], [7], [8] and on LL part of each segment singular value decomposition (SVD) [9]. The ownership share is produced as an output. An image, secret keys, and ownership share are used at the detection side.

The next method selected is “Discriminative and robust zero-watermarking scheme based on the completed local binary pattern for authentication and copyright identification of medical images” [10]. This method uses a low-pass Gauss filter, vectors with pixel approximation, and the *completed local binary pattern* method [11]. As input, it used a grayscale image and a binary watermark. The output is an ownership share.

The last method examined is *A QR Code Based Zero-Watermarking Scheme for Authentication of Medical Images in Teleradiology Cloud* [12]. It uses as input a grayscale image, a binary watermark, two secret keys, and the block size and number of iterations of the Contourlet transformation [13]. Low-frequency sub-bands are divided into blocks, and *singular value decomposition* [9] is applied.

## Results

To demonstrate the outcome of our research, we have selected representative of various watermarking methods with different ways of watermark insertion and representing a different class of zero-watermarking algorithms commonly used in medicine. As will be observable from the results below, all zero-watermarking algorithms except one provide similar results. Due to our long-term cooperation with Masaryk Memorial Cancer Institute, the algorithms examined were used on (anonymized) CT images of patient with tumor recommended by physicians from this institute.

During our testing of zero-watermarking methods, we conducted a lot of simulations and comparisons.

**Fig. 3:**
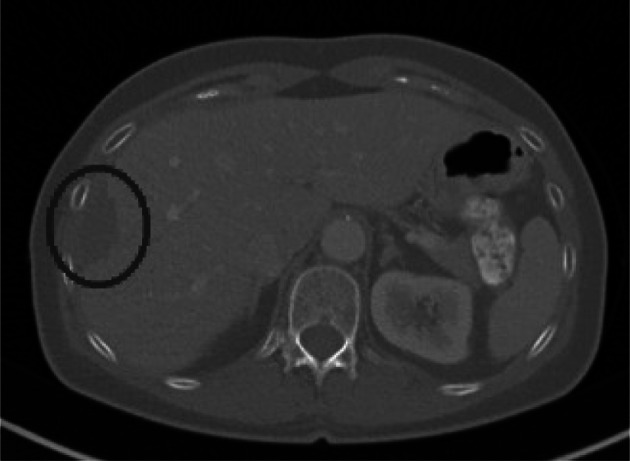
Part of original CT image with marked tumor

In the first phase, we focused on observing the behavior of individual zero-watermarking methods in case of modifying the secured image (see Fig. 3).

**Fig. 4:**
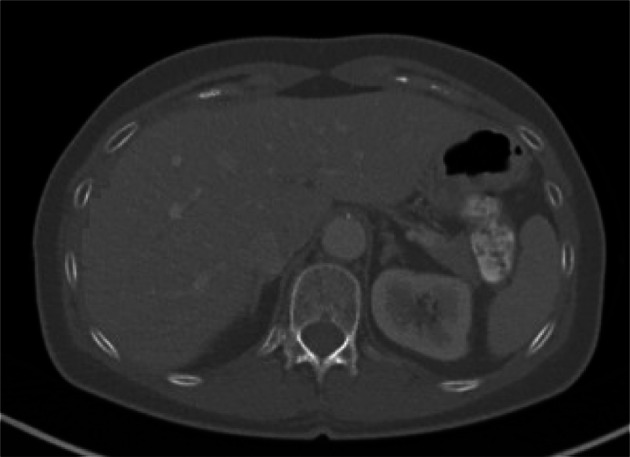
Part of CT image with the graphically removed tumor

**Fig. 5:**
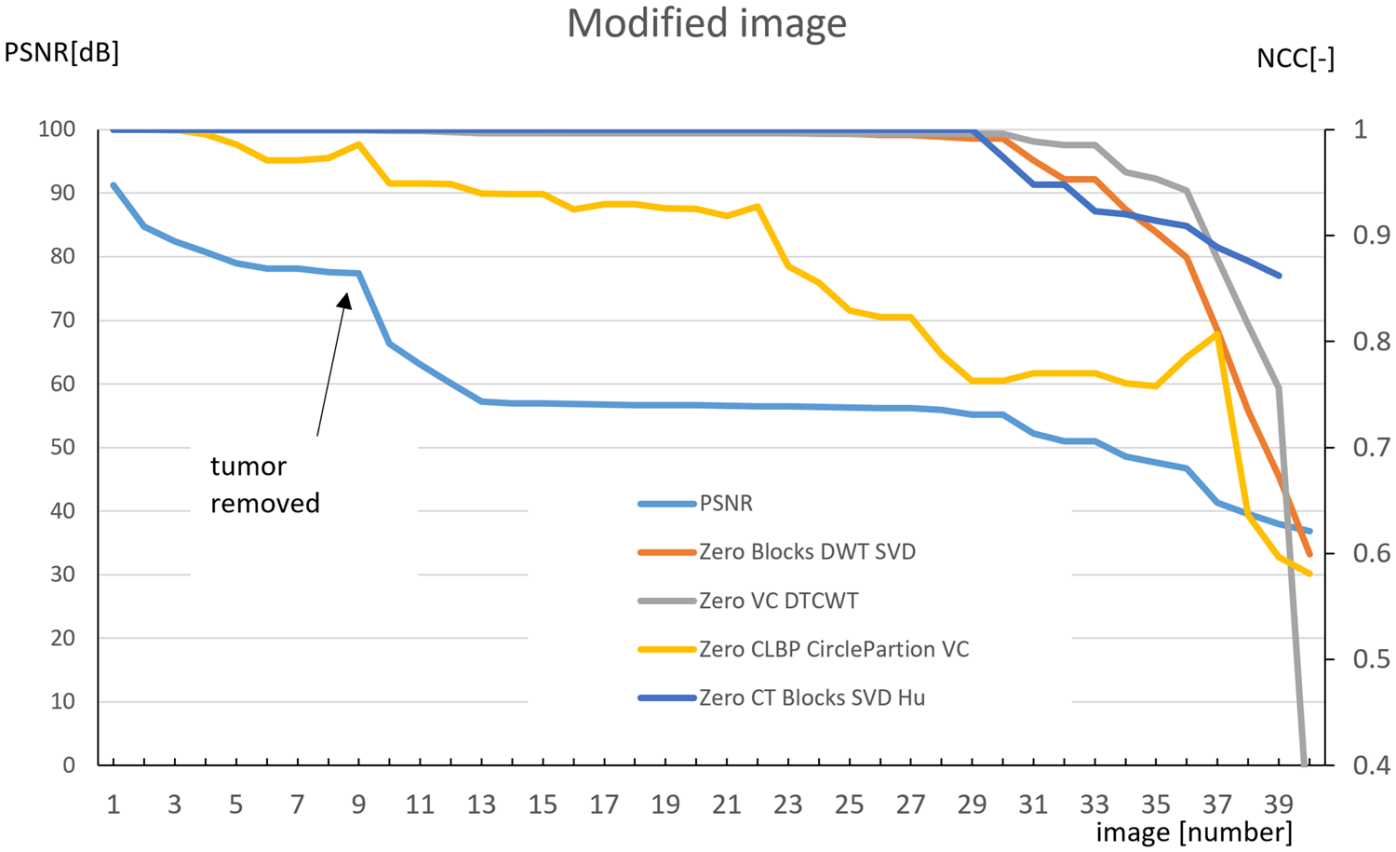
Attacking the security of zero-watermarking methods by modifying the watermarked image

The result of this phase is shown as a graph in Fig. 5. Increasing the graphical modification of the secured image (from image numbers 2 to 40) decreases the PSNR (a metric of the similarity of the secured and modified images). Firstly, we gradually graphically removed a tumor (see Fig. 4); then, we made further gradual destructive changes in the medical image.

During this progress, we observed the behavior of the particular watermarking methods. In other words, we reduced the resemblance of the secured image and the modified image from which watermark is extracted (the PSNR), and we observed the similarity of the embedded and extracted watermark NCC (normalized cross correlation [14]). From the chart at Fig. 5, it is clear that the individual methods (except for zero CLBP [10]) can relatively correctly extract the watermark, even if the image is largely modified. In case of unauthorized modification of the secured image, it is, therefore, possible to prove its authorship by using suitable methods of zero watermarking. It is also possible to ensure integrity in case of modification because the NCC (normalized cross correlation) of modified and original images is never exactly equal to 1. For example, watermark extracted from modified image number 2 has NCC = 0.99978.

**Fig. 6:**
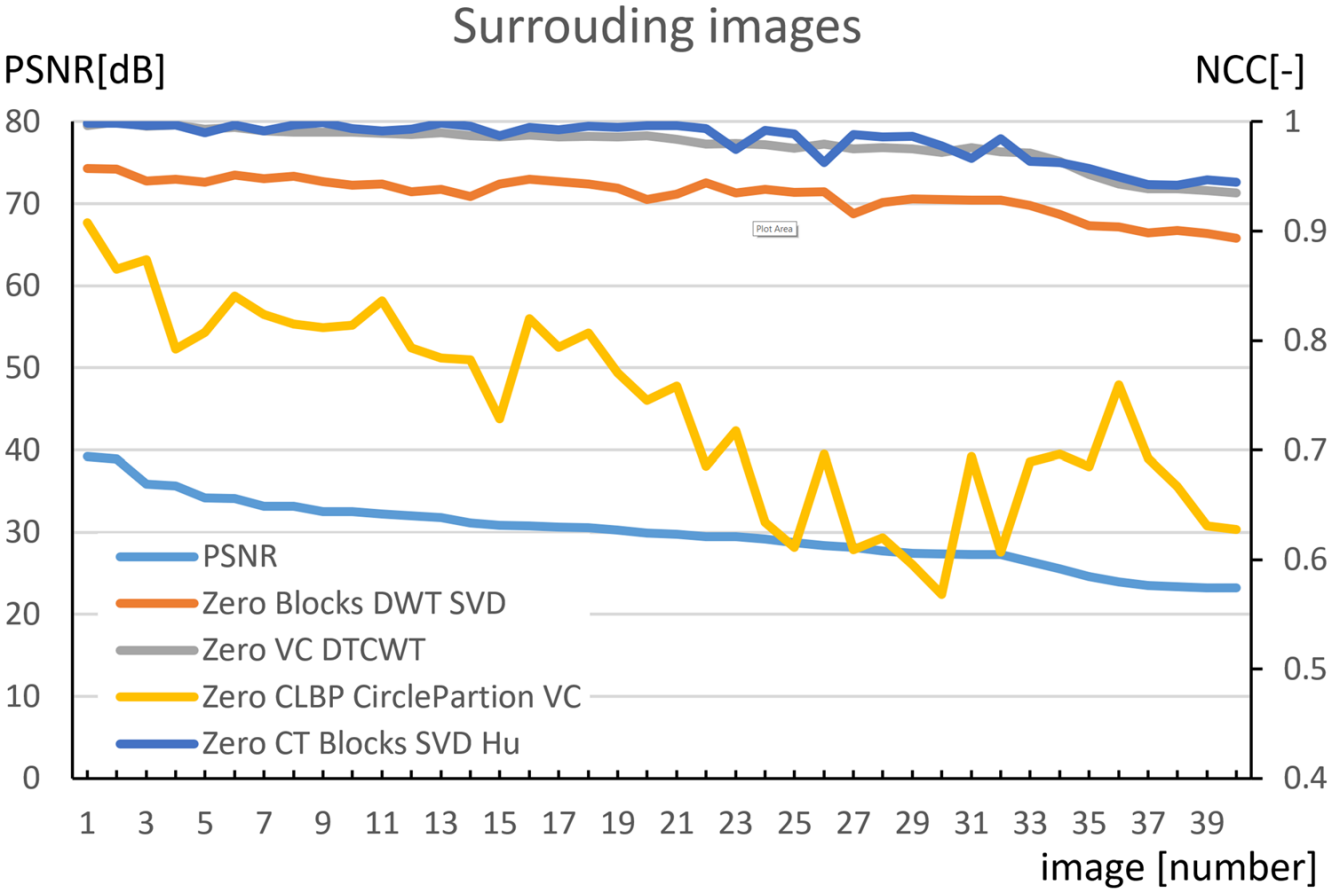
Attacking the security of zero-watermarking methods by replacing the watermarked image with surrounding CT images

In the next phase, we compared characteristics of watermarking methods in the instance of exchanging watermarked images with neighboring CT images of the same patient. The graph in Fig. 6 shows that the similarity of the secured and changed images (from which the watermark is extracted) is low (PSNR < 40 dB; medical images are ordered from higher PSNR to lower). Nevertheless, individual methods of zero watermarking display relatively high NCC values between inserted and extracted watermarks. This means that the watermarks are quite similar.

These methods are therefore inappropriate to protect authorship when exchanging the watermarked image with neighboring images. This is because it is not possible to recognize if the medical image was only modified (and is owned by his author), or if it has been replaced with a different image of the same patient.

In the final stage, similarly to the previous phase, where we exchanged a former secured image with neighboring images, we exchanged a formerly secured image with an image of the same body part of a totally different patient. As in the previous phase, similarity of the secured and exchanged images is low (PSNR < 30 dB; medical images are ordered from higher PSNR to lower), the NCC values of the inserted and extracted watermarks were rather high, despite the low similarity of the secured and changed (attacked) images.

**Fig. 7:**
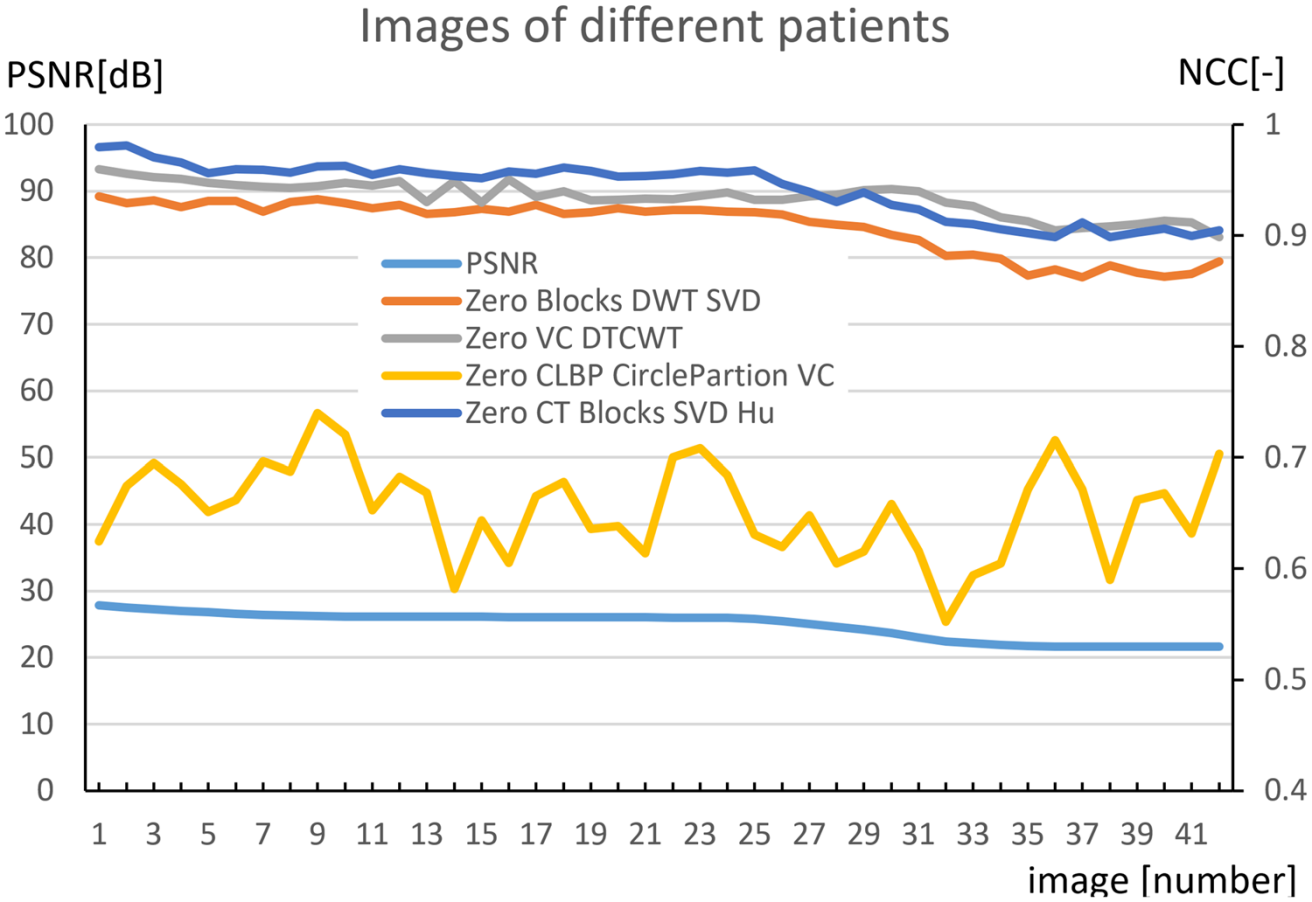
Attacking the security of zero-watermarking methods by replacing a watermarked CT image with images of different patients

It is obvious from Fig. 7 that watermarking methods do not respond well even to this drastic form of image substitution and thus are not suitable for securing authorship.

The NCC itself is only an image similarity measure. If the NCC of inserted and extracted pair of watermarks is high, it still does not guaranty that the image the watermark was extracted from is a derivative of the original. It only expresses our belief or plausibility of being so. Currently, NCC above 0.7 is taken as the touchstone whether it is tested image suspected to be a derivative of the protected one. Proper determination of the threshold or possible two (soft and hard) thresholds is subject to further study. The Zero CLBP CliclePartition VC method comparing with others seems to be the much more sensitive in distinguishing modified picture from a completely different one.

## Analysis of Applicability

The presented approach represents a suitable solution for protecting the integrity, and at the same time, enabling controlled sharing in medical imaging. It allows the detection of breaches of personal data protection obligations. For the case of dispute over the ownership of the imaging study, the owner’s identity can be invisibly inserted into the image. It enables one to distinguish individual copies of the imaging study (by embedding specific part of the watermark per legitimate user), and this way identify the potential originator of the illegal copy. Some highly sensitive information may remain hidden from unauthorized participants.

The important thing is that some modifications or the addition of trial relevant information are permissible. All the changes can be then (for the assertion of authenticity) verified by the entitled party. The schema can distinguish the level of modification, or whether any took place at all, and this way provides the desired trade-off in the protection of shared medical content. Just binary information provided by digital signature techniques is its main shortcoming when compared with watermarking.

Embedded hidden information can indicate the ownership, can contain some object-specific information like patient identity (pseudo-identity), or convey conditions of legal usage (preventing illegal redistribution) via embedding a unique mark for each legitimate user. The secret key, which is necessary to recover the embedded information, can be shared within the research community or be under the sole control of the data owner.

For the introduced usage scenario, an appropriate watermarking schema provides the desired flexibility. The advantage of watermarking over the digital signature in the collaborative environment, where the medical images are subject to legitimate modification (highlighting of the significant region, labeling of notable feature, etc.), is visible.

## Conclusion

This article deals with testing zero-watermarking methods to ensure the authorship and integrity of medical image data. This protection is necessary, for example, when sharing images for educational or scientific purposes. Securing medical image data with zero-watermarking methods has many undisputed benefits: it does not modify secured data, and the participation of trusted authority, the use of timestamps, and further processing of secured data are all allowed—they are not encrypted or modified.

We conducted three types of tests as simulations of attacks: modifying the secured image itself, replacement of the secured image by neighboring images in CT studies, and replacement of the secured image by images of the same part of the body, but a different patient.

We concluded that zero watermarking is suitable for checking the integrity of digital medical images, but it is not usable for securing their authorship. This is because generally, zero watermarking is not able to recognize differences between an image modified by an attack intended to steal its ownership and a completely different image.

The zero-watermarking algorithms used for this task generate too many false-positive alarms. Zero watermarking is not able to reliably enough distinguish a modified image belonging to the author from somebody else’s similar image. Especially in the medical field, it is not capable of distinguishing images of the same part of the body originating from two different patients.

On the other hand, zero-watermarking algorithms proved a good reflection of the degree of image modification, so they probably could be used as a measure of image similarity. The utilization of zero-watermarking algorithms for this purpose needs further study and testing.
